# Quantification of intra and inter-ventricular dyssynchrony in Ebstein's anomaly using cardiovascular magnetic resonance myocardial feature tracking

**DOI:** 10.1186/1532-429X-16-S1-O108

**Published:** 2014-01-16

**Authors:** Michael Steinmetz, Sophie-Charlotte Alt, Shelby Kutty, Jan M Sohns, Christina Unterberg-Buchwald, Thomas Paul, Gerd Hasenfuss, Joachim Lotz, Pablo Lamata, Andreas Schuster

**Affiliations:** 1 Department of Pediatric Cardiology and Intensive Care Medicine and Heart Research Center, Georg-August-University, Göttingen, Germany; 2 University of Nebraska College of Medicine and Children's Hospital, Medical Center, Omaha, Nebraska, USA; 3 Institute for Diagnostic and Interventional Radiology, Georg-August-University, Göttingen, Germany; 4 Department of Cardiology and Pneumology and German Centre for Cardiovascular Research, Georg-August-University, Göttingen, Germany; 5 Department of Computer Science, University of Oxford, Oxford, UK; 6 Dept. of Biomedical Engineering, St Thomas' Hospital, King's College, London, UK

## Background

Ebstein's anomaly consists of varying degrees of tricuspid dysplasia and displacement into the right ventricular (RV) cavity, along with changes in ventricular function predominantly affecting the RV. Measuring alterations in contraction pattern and dyssynchrony may provide insights into ventricular performance in this disease. We sought to quantify right and left ventricular (RV and LV) deformation and dyssynchrony in a cohort of patients with Ebstein's anomaly in comparison with normal controls using cardiovascular magnetic resonance myocardial feature tracking (CMR-FT).

## Methods

Twenty-six patients with uncorrected Ebstein's anomaly and 10 normal controls were prospectively studied. LV and RV global longitudinal myocardial strain (GLSLV and GLSRV) and time to peak strain (TPKLV and TPKRV) were calculated from the 4-chamber cine CMR images. Intraventricular dyssynchrony (IntraVD) was calculated as the difference between the segments with the longest and shortest TPK divided by the global TPK value of the respective ventricle: (IntraVD) = TPKlongest-TPKshortest/TPK global. The ratio of global TPKRV and TPKLV was measured as interventricular dyssynchrony (InterVD).

## Results

Mean patient age was 28 ± 15 (69% male sex). GLSRV was significantly reduced in patients (-15.7 ± 5.2) as compared to controls (-21.1 ± 2.9, p = 0.001), while there was no significant difference in GLSLV or global TPKRV and TPKLV between patients and controls (p > 0.05, Table 1). The Ebstein's RV showed a significantly higher degree of IntraVD (0.96 ± 0.39) compared to the Ebstein's LV (0.74 ± 0.37, p = 0.03), and compared to the RV (0.55 ± 0.24) and LV (0.65 ± 0.29) of healthy controls (p < 0.05). There was no significant difference between the LV IntraVD of Ebstein's patients and the LV and RV IntraVD of controls (p > 0.05). There was also no significant difference in InterVD between the Ebstein's group (1.03 ± 0.2) and controls (1.02 ± 0.12, p = 0.99).

**Table 1:** Strain values and times to peak in Ebstein's anomaly and normal controls.

Mean ± Standard Deviation	LV		RV	
	GLS [%]	TPK GLS [ms]	GLS [%]	TPK GLS [ms]
Ebstein's Anomaly	-17.2 ± 4.3	330 ± 58	-15.7 ± 5.2	338 ± 78
Volunteers	-17.7 ± 4.3	336 ± 36	-21.1 ± 2.9	338 ± 26

## Conclusions

These results demonstrate RV intraventricular dyssynchrony and reduced global RV strain in Ebstein's anomaly that can be derived by CMR myocardial feature tracking. Larger studies are needed to understand the significance of these parameters in the disease progression and their clinical implications.

## Funding

German Centre for Cardiovascular Research (DZHK Partner Site Göttingen).

